# Preconditioning 2D Integer Data for Fast Convex Hull Computations

**DOI:** 10.1371/journal.pone.0149860

**Published:** 2016-03-03

**Authors:** José Oswaldo Cadenas, Graham M. Megson, Cris L. Luengo Hendriks

**Affiliations:** 1 School of Systems Engineering, The University of Reading, Reading, RG6 6AX, United Kingdom; 2 School of Electronics and Computer Science, University of Westminster, London, W1W 6XH, United Kingdom; 3 Department of Information Technology, Uppsala University, Box 337, SE-751 05 Uppsala, Sweden; Fondazione Edmund Mach, Research and Innovation Centre, ITALY

## Abstract

In order to accelerate computing the convex hull on a set of *n* points, a heuristic procedure is often applied to reduce the number of points to a set of *s* points, *s* ≤ *n*, which also contains the same hull. We present an algorithm to precondition 2D data with integer coordinates bounded by a box of size *p* × *q* before building a 2D convex hull, with three distinct advantages. First, we prove that under the condition *min*(*p*, *q*) ≤ *n* the algorithm executes in time within *O*(*n*); second, no explicit sorting of data is required; and third, the reduced set of *s* points forms a simple polygonal chain and thus can be directly pipelined into an *O*(*n*) time convex hull algorithm. This paper empirically evaluates and quantifies the speed up gained by preconditioning a set of points by a method based on the proposed algorithm before using common convex hull algorithms to build the final hull. A speedup factor of at least four is consistently found from experiments on various datasets when the condition *min*(*p*, *q*) ≤ *n* holds; the smaller the ratio *min*(*p*, *q*)/*n* is in the dataset, the greater the speedup factor achieved.

## Introduction

Computing the convex hull on a set of *n* 2D points is a first computational step to many geometric algorithms [1]. It has many practical applications; in ecology [2], neuroscience [3], computer vision [4] and palaeontology [5] to name a few. Most known convex hull algorithms are of time complexity *O*(*nlogn*) [6]; these algorithms are general in the sense that they do not impose any restriction on the order in which points are considered. Linear complexity (*O*(*n*)) time algorithms do exist but require a set of points that are ordered in some way; for example [7] requires an order where the points form a simple polygonal chain. Such orderings are not always easy to produce given the process of data collection. Regardless of the time complexity of an algorithm, reducing the set of *n* points down to a set of *s* ≤ *n* points would result in faster computations, provided that the smaller set preserves the convex hull of the original (bigger) set, and provided that the time taken to perform the reduction offsets the cost of preprocessing *n* − *s* points for any convex hull building algorithm. This kind of reduction is often used as the first step in practical implementation of convex hull algorithms seeking to reduce execution time [8]. Here, a new and surprisingly simple method to perform a reduction of 2D points bounded in a box of size *p* × *q* with integer coordinates is empirically analyzed [9]. The method is based on a proposed algorithm that exhibits three distinct advantages over other methods.

- First, we prove that under the condition *min*(*p*, *q*) ≤ *n* the method is linear and can be applied before any known 2D convex hull algorithm.
- Second, no explicit sorting of points is required.
- Third, by construction, the reduced set of points forms a simple polygonal chain and hence directly prepares the points for linear convex hull algorithms such as the one of Melkman [7].

The second bullet point is significant because common reduction methods require an explicit call to a sorting procedure of the points along a particular direction [8].

First we measure the amount of reduction of points achieved by the proposed method; we find higher amounts of reduction than the ones obtained by the most common method [8]. We also show, through experimental evaluation, that the method makes faster convex hull computations for both linear and non-linear algorithms. Experiments were carried out on four different datasets having different *p*/*n* ratios (assuming *p* ≤ *q*). In order to benchmark the speedup benefit of the proposed preconditioning method, the experiments use the most common convex hull computational algorithms readily available in the CGAL computational geometry library in order to build the final hull [10] (and briefly from OpenCV [11]). Since our method directly forms a polygonal chain, for completeness, we also apply it to the method of Melkman, which is not possible with the most common method [8]. We have also included results using the convex hull algorithm from Chan [12].

## Materials and Methods

### Proposed algorithm

**Fig 1:**
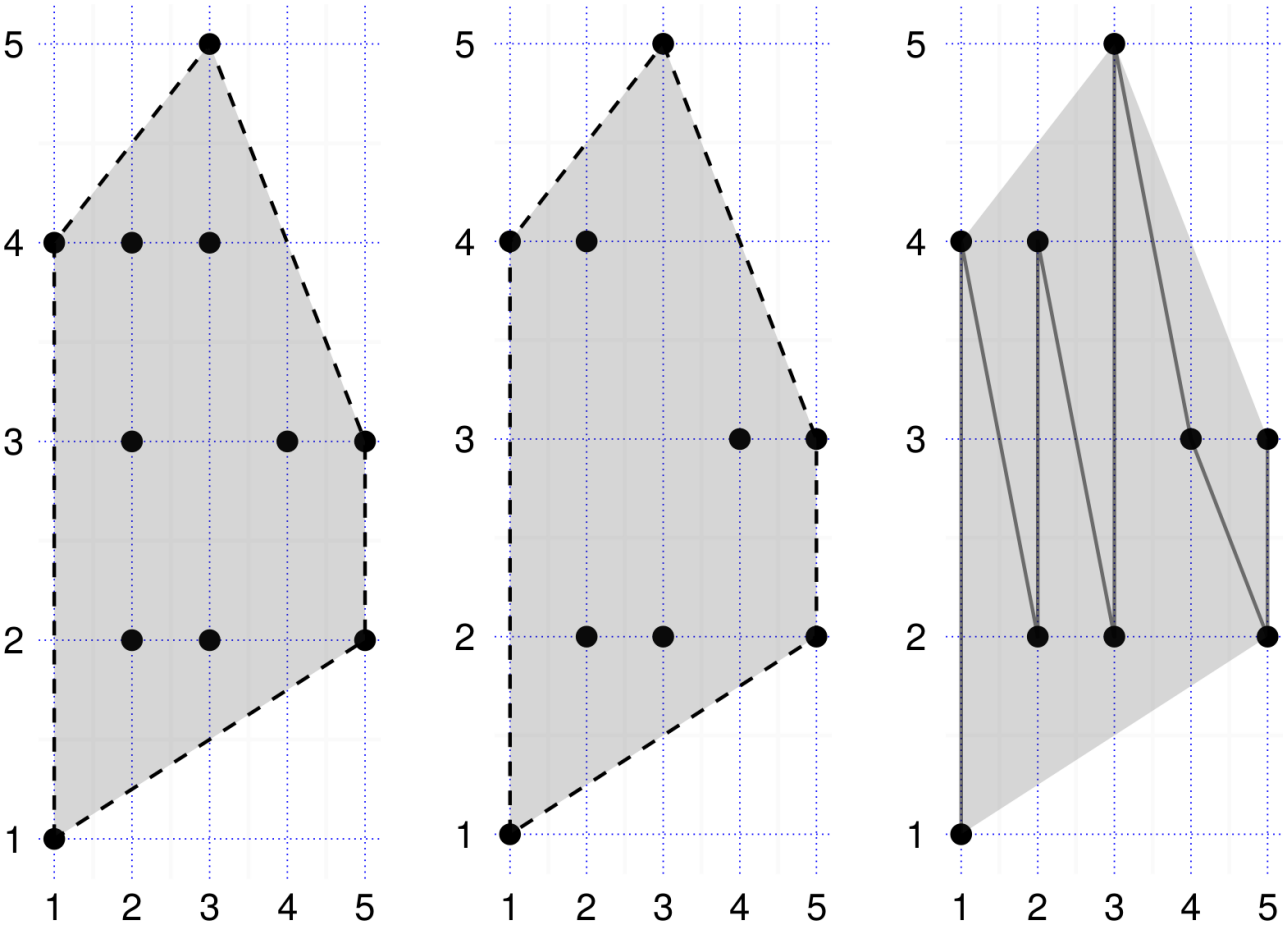
A small example of 2D points with integer coordinates. Left: (*x*, *y*) integer points on a 2D grid with *p* = 5. Center: Points with minimum and maximum *y* values for each *x* coordinate. Right: A polyline.

Assume a 2D box with sides *p* × *q*, with integer points whose *x*, *y* coordinates are in the range [1, …, *p*], and [1, …, *q*] respectively. Without loss of generality, assume *p* ≤ *q*. As a small example consider the set of (*x*, *y*) points on the left of Fig 1, given in any order as an array *P*.

Assume an array *L* of *p* entries as points, with each point initialized to (*q* + 1,−1). The proposed algorithm is presented in pseudo-code in Algorithm 1 listing. After all *n* points of *P* have been processed by the routine in Algorithm 1, *L* = [(1, 4), (2, 4), (2, 5), (3, 3), (2, 3)]. *L*[1] = (1, 4) since, *y* = 1 is the minimum point (min); and *y* = 4 is the maximum (max) point for column *x* = 1. This reduced set is shown in the center of Fig 1. Intuitively the convex hull on the left is the same as the convex hull on the center of the figure. Since local convexities of the boundary points are conserved and collinear points are removed we need only to consider the min and max points on an *x* (or *y*) dataset when deriving a convex hull [6].

**Algorithm 1** Reduction of points

**Input:** An array *P* of *n* points with (*x*, *y*) coordinates, *x* ∈ [1…*p*] and *y* ∈ [1…*q*]

**Require:** Initialize *L*[*i*] = (*q*+1,−1), ∀*i* ∈ [1, *p*]

**Output:** An array *L* of *p* entries with each entry either point (*u*, *v*) or (*q*+1,−1)

 1: **foreach** *point* **in** *P* **do**

 2: *x*_*i*_, *y*_*i*_ = *point*

 3: *u*, *v* = *L*[*x*_*i*_]

 4: *u* ⇐ *min*(*y*_*i*_, *u*)

 5: *v* ⇐ *max*(*y*_*i*_, *v*)

 6: *L*[*x*_*i*_]⇐(*u*, *v*)

 7: **end for**

#### Building a polyline

Scanning *L* along *x* builds a simple polygonal chain, since joining all points of the reduced set of points *s* ≤ *n* creates edges that do not intersect. For each valid point in *L* (one different to (*q*+1,−1)) joining *u* to *v* (min to max), and then from *v* (max) to *u* (min) of the next valid point, forms a simple polygonal chain. We formalize this principle with the following lemma.

**Lemma 1.** *For a bounding box of m* = *p* × *q* *points with integer coordinates, and given an array L with entries* (*u*_*i*_, *v*_*i*_), *i* ∈ [1…*p*] *so that* 1 ≤ *u*_*i*_, *v*_*i*_ ≤ *q are the minimum and maximum points respectively at x coordinate i, there exists a simple chain joining all the minimum and maximum points*.

*Proof (by construction)*. The chain is formed by scanning entries of *L* along *i* = 1, …, *p*. Each entry *i* of *L* contains one or two points or no points of the bounding box. Let’s refer to an entry (*u*_*i*_, *v*_*i*_) of *L* such that 1 ≤ *u*_*i*_, *v*_*i*_ ≤ *q* as corresponding to a valid point. An entry (*u*_*i*_, *v*_*i*_) = (*q* + 1,−1) corresponds to a column in the bounding box with no valid points. An entry *i* in *L* with no valid points is skipped. If the entry *i* has a single valid point (*u*_*i*_ = *v*_*i*_), that point is kept. Two valid points in an entry *i* are joined with an edge that runs from (*i*, *u*) to (*i*, *v*). The whole chain is formed by connecting edges in an entry *i* (or single point in entry *i* or skipping entry *i*) to edges in adjacent entries (or single point in adjacent entry or skipping adjacent entry) until all valid points are connected. Adjacent entries *i* and *i* + 1 are connected by an edge from (*i*, *v*) to (*i* + 1, *u*) or from (*i*, *v*) to (*i* + *k*, *u*) where entries *i* + 1, …, *i* + *k* − 1 have no valid points. This creates a simple chain covering all the valid points in *L* since no edges intersect.

#### Quick analysis

The routine of Algorithm 1 visits each point of *P* once, therefore *L* is built in *O*(*n*) time. It can be argued the running time is within *O*(*n* + *p*) but as we restrict points to the case *p* ≤ *n*, then the running time is *O*(*n*). Scanning *L* to recover valid points builds the polygonal chain, Lemma 1, takes *O*(*p*) time and provided that *p* ≤ *n* the whole method of building the polygonal chain takes *O*(*n*) time. As the maximum number of valid points that remain after the reduction by Algorithm 1 is *s* = 2*p* then a potential reduction of the number of original points is given by the factor $1-\frac{2p}{n}$. Clearly, the smaller the ratio *p*/*n* the greater the reduction that will be achieved. For the case *p* > *n* this paper does not make any claim of any advantages for Algorithm 1 for two reasons. Firstly, the algorithm takes *O*(*n* + *p*) time and secondly, in practice it corresponds to sparse datasets, where few points would be removed, and thus it is not worth attempting any preconditioning of points.

#### Correctness of Algorithm 1

See Appendix.

### Common method to reduce a set of 2D points

A simple algorithm to compute the convex hull on a set of 2D points is presented in [8]. In the general case, the algorithm has a worst case time complexity of *O*(*nlogn*), mainly due to an explicit sort on points based on their *x* coordinate. It is composed of three steps, with a first step being a pre-processing procedure, with running time *O*(*n*), that finds extreme points from where a reduction of points is made before attempting to build the final convex hull. The preprocessing follows by determining the minimum and maximum values along *x* and *y* coordinates. With these points, four or fewer external regions are formed. All the points found inside these external regions are discarded since they cannot belong to the convex hull. Applying this method to the small example on the left of Fig 1, three external regions are formed (outside the convex hull and inside the box). All points internal to the pentagon (convex hull of the figure, shaded) can be discarded. In this trivial example, this method has not left any points to be further considered. In general, it is reported that for large values of *n* the number of points is reduced to less than half [8].

### Proposed method to reduce a set of 2D integer points

Determining the minimum and maximum values along the *x* coordinate gives *p* as *p* = *x*_*max*_ − *x*_*min*_ + 1, and thus, resolving whether the condition *p* ≤ *n* holds or not, carries the same time cost *O*(*n*) as the first step of the common preconditioning method in [8]. This includes any translation of points if necessary. We propose the following preconditioning method.

1. Determine *min*(*p*, *q*) and *n* from the dataset. This takes *O*(*n*) time.
2. Check the condition *min*(*p*, *q*) ≤ *n*. If the condition holds, precondition points by Algorithm 1 (if *p* > *q* Algorithm 1 is applied with array *L* along the *y* coordinate instead of *x*). Execute Step 3 below with the remaining *s* points after the reduction. If the condition does not hold, skip Algorithm 1 and execute Step 3 below with the original *n* points. This step also takes at most *O*(*n*) time.
3. Call any convex hull algorithm to build the final hull.

The overall effect is that determining whether to apply the proposed method and applying the method will not make the computation of a convex hull any worse regardless of the chosen convex hull algorithm. From the previous analysis it follows that for datasets with ratios *p*/*n* < 1/4, the reduction of points will be of more than half.

## Results

### Empirical evaluation on four datasets

A quantitative evaluation on four datasets is presented. The datasets are: a dataset with random numbers referred to as the synthetic dataset, a dataset with points densely distributed in a box, a typical image dataset, and a dataset with a low density of points in a box or sparse dataset. The first three datasets correspond to cases where the condition *p* ≤ *n* holds. The points in the sparse dataset present the condition *p* > *n*.

**Fig 2:**
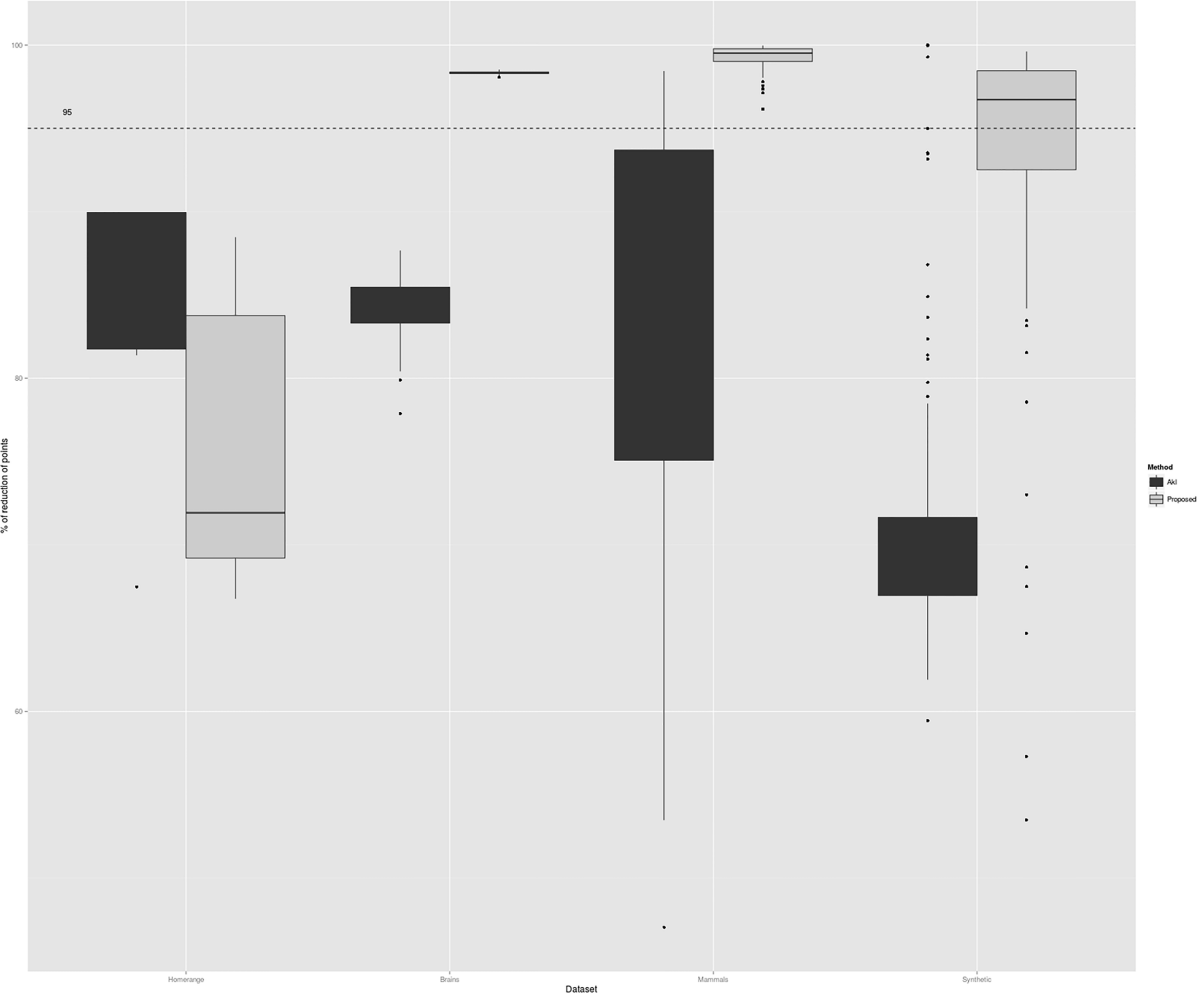
Percentage of reduction of points of four datasets of 2D points with integer coordinates. The result of two methods of reductions are shown: The one of Akl and Toissant as presented in [8] and the one proposed here.

The amount of reduction of points that results using the most common preconditioning method of Akl and Toussaint [8] and the reduction that results by applying the proposed preconditioning method are presented. The result of the reduction of points is shown in Fig 2. It is clear that Akl and Toussaint’s method gives reductions of over half the points as claimed [8]. As previoulsy noted, the proposed method struggles to reduce points substantially under the condition *p* > *n*, as seen in the sparse dataset. The figure also shows that the preconditioning method proposed here gives a reduction in the number of points of over 95% when the condition *p* ≤ *n* holds; this reduction is clearly greater than the reduction obtained by the method of Akl and Toussaint for this conditions on the same datasets. A greater reduction compared to Alk and Toissaint’s, like the observed in Fig 2, is useful in practice if the time to perform this reduction plus the time to compute the convex hull on the remaining smaller set of points gives an overall speedup running factor. This is examined next.

#### Synthetic dataset

**Fig 3:**
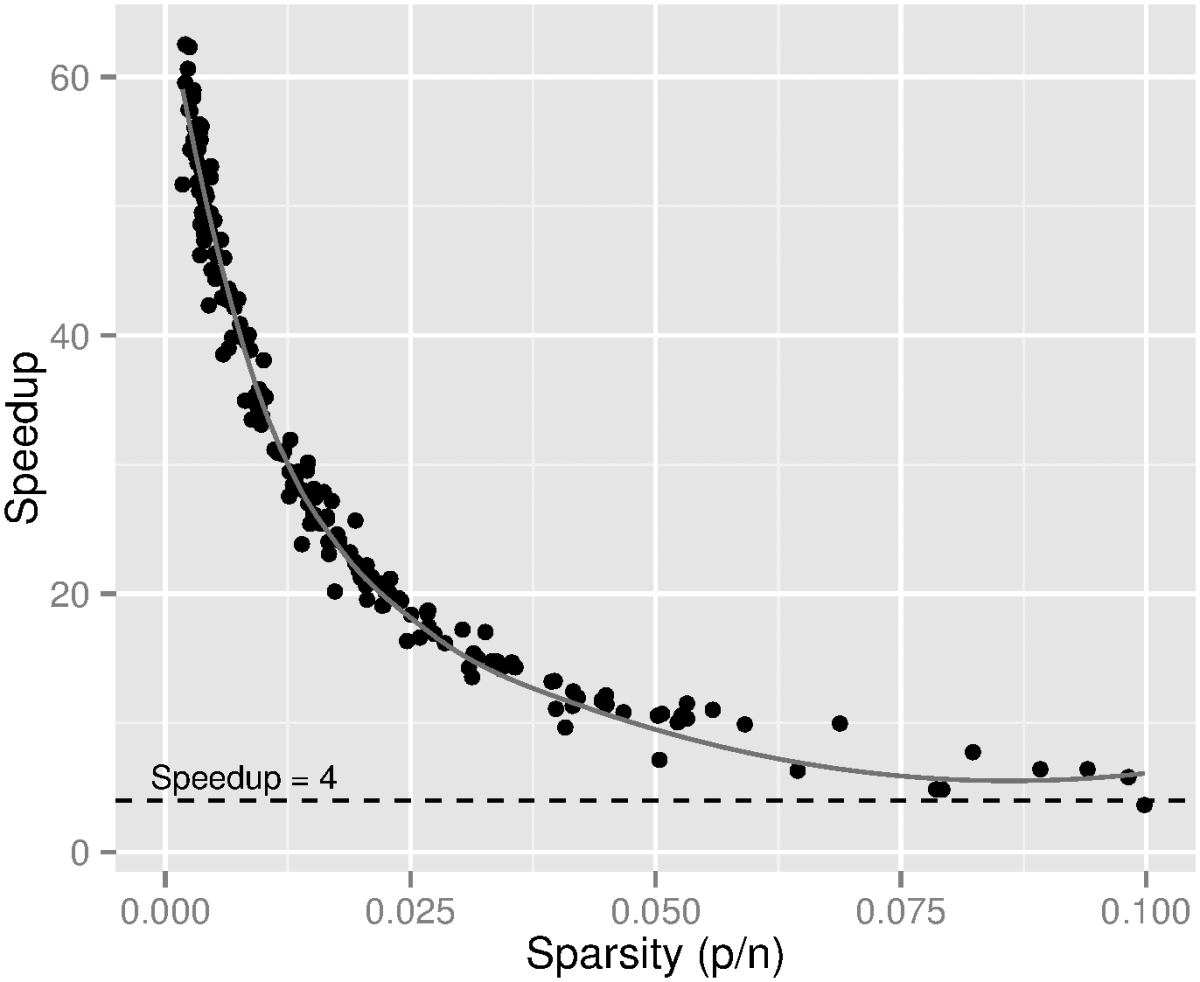
Speedup factor as a function of *p*/*n* for a random set of circles and superellipses. A set of *n* points in a box of *p* × *q* were preconditioned first by the method proposed here and the convex hull found by the algorithm in [8] as available from CGAL [10].

For the first dataset many circles and super-ellipses were generated inside *p* × *q* boxes of typical image sizes (from under 1 Mpixel to 40 Mpixels) each having *n* random points. For each circle or super-ellipse the convex hull was computed using the algorithm (and preconditioning method) in [8] as available from CGAL and the execution time annotated as *t*_*n*_. The proposed preconditioning method was applied to each circle and superellipse to reduce the original number of *n* points to *s* points and the execution time for this reduction was annotated as *t*_*r*_. The execution time to get the convex hull on each reduced set was annotated as *t*_*s*_ (including the time to extract valid points from array *L*). A speedup factor was then computed as $\frac{t_{n}}{t_{r}+t_{s}}$. This speedup factor is seen in Fig 3 as a function of *p*/*n* (referred to as sparsity). A speedup factor of at least four is consistently observed in Fig 3 when computing the convex hull with the proposed preconditioning method. For this random dataset, the condition *p* ≤ *n* always holds.

#### Dense dataset

**Fig 4:**
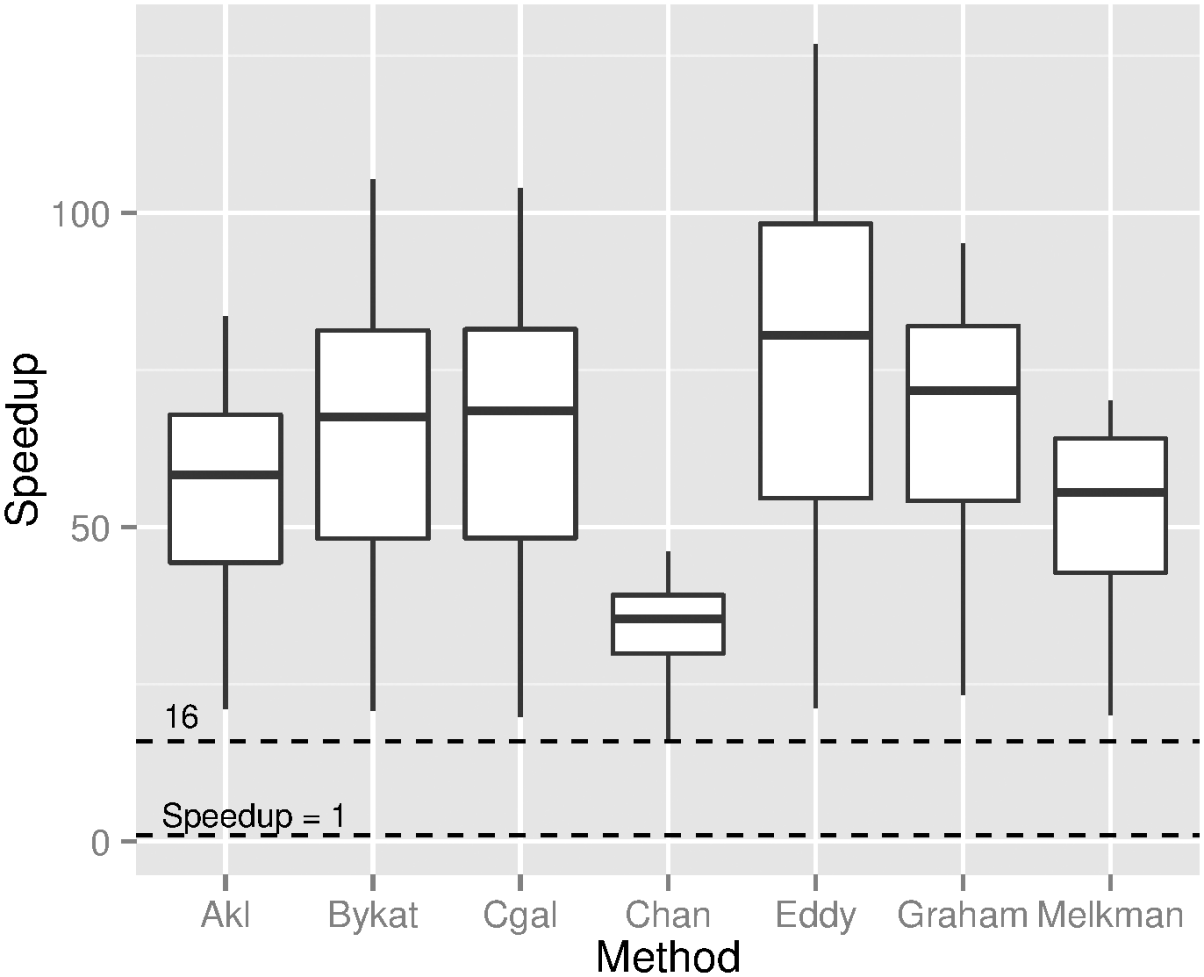
Speedup factor for a dense dataset. The points of a dataset of 13 mammals were first preconditioned with the method proposed here and then the convex hull was computed with seven algorithms.

For the second dataset we post-processed a dataset of a 3D point cloud representing complete models of each of 13 large-bodied mammals [5] (data available from http://www.animalsimulation.org). A series of 2D projections along one axis on a plane with integer coordinates was performed for each mammal’s 3D model to generate very dense datasets; these have *n* of up to 8 million points. We used 2D boxes of size *p* × *q* with *p* ranging from 1024 to 10320. A speedup factor of at least a factor of 16 is consistently observed in Fig 4 when computing the convex hull with the proposed preconditioning method. Note that the final hull was built by six algorithms available from CGAL, as well as Chan’s algorithm (algorithm CGAL is without the preconditioning in [8]). For this dataset the condition *p* ≤ *n* always holds.

#### Typical image dataset

**Fig 5:**
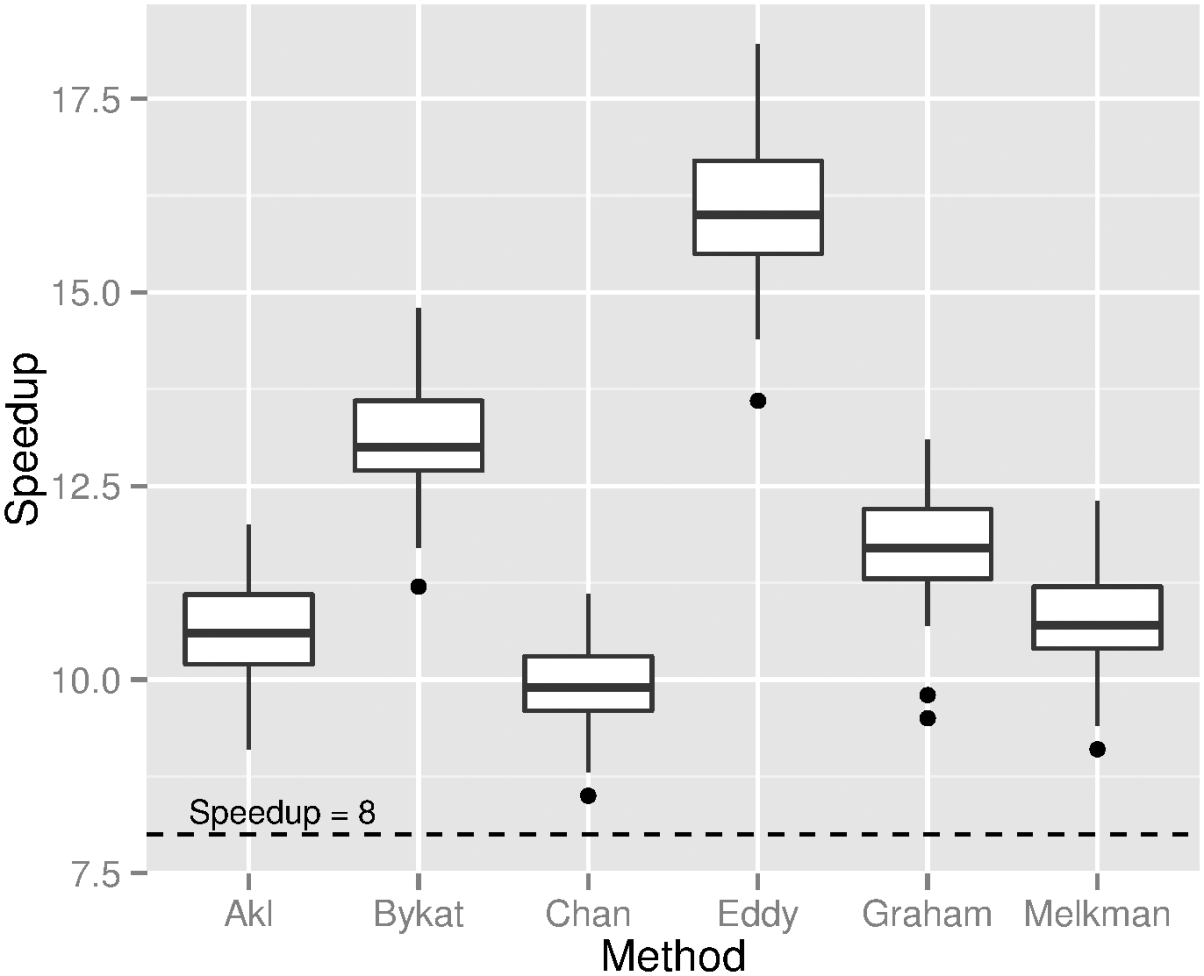
Speedup factor for a typical image dataset. The points of a dataset of 49 brain images were first preconditioned with the method proposed here and then the convex hull was computed with six algorithms.

For the third dataset we took a collection of 49 brain MRI midsagittal planes. The intercranial volume in each MRI image was manually segmented [13]. We extracted the midsagittal plane as a 2D binary image, with a pixel size of 0.94*mm* × 3.00*mm*. For this dataset, a speedup factor of at least a factor of eight is consistently observed when computing the convex hull with the proposed preconditioning method (Fig 5). For this dataset the condition *p* ≤ *n* always holds.

#### Sparse dataset

**Fig 6:**
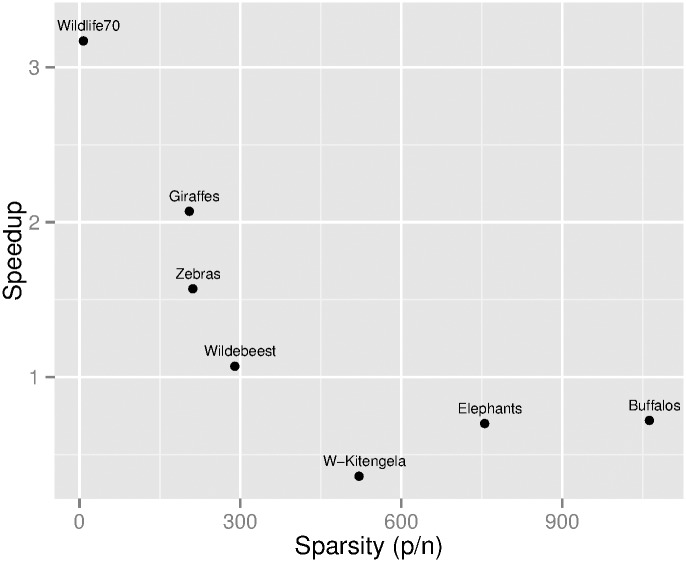
Speedup factor as a function of *p*/*n* for a sparse dataset. The points of seven homerange datasets were first preconditioned with the method proposed here and then the convex hull was computed by Chan’s algorithm.

For the fourth dataset we have used seven recordings of 2D spatial distribution of wildlife in Kenya [14]. These datasets annotate the area where animals move around in search of food or their homerange. The data annotate area positions using a 2D Cartesian coordinate referred to as Universal Transverse Mercator system (UTM) that we have resolved to distances of 1 meter. This results in cases where the homerange area is bound to *p* values close to a million meters. Each dataset recorded at most 120 thousand positions and as such we have a sparse case where condition *p* ≤ *n* does not hold. The potential acceleration obtained by using the proposed preconditioning method for these datasets is shown in Fig 6; Chan’s convex hull algorithm was used to build the final hull. The graph shows that the closer to 1 the ratio *p*/*n* is, the better the speedup factor achieved. However, for this case, *p* > *n*, the proposed preconditioning method is not worth using as we have already noted; as expected, no practical advantage by reducing the number of points was achieved.

## Discussion

**Fig 7:**
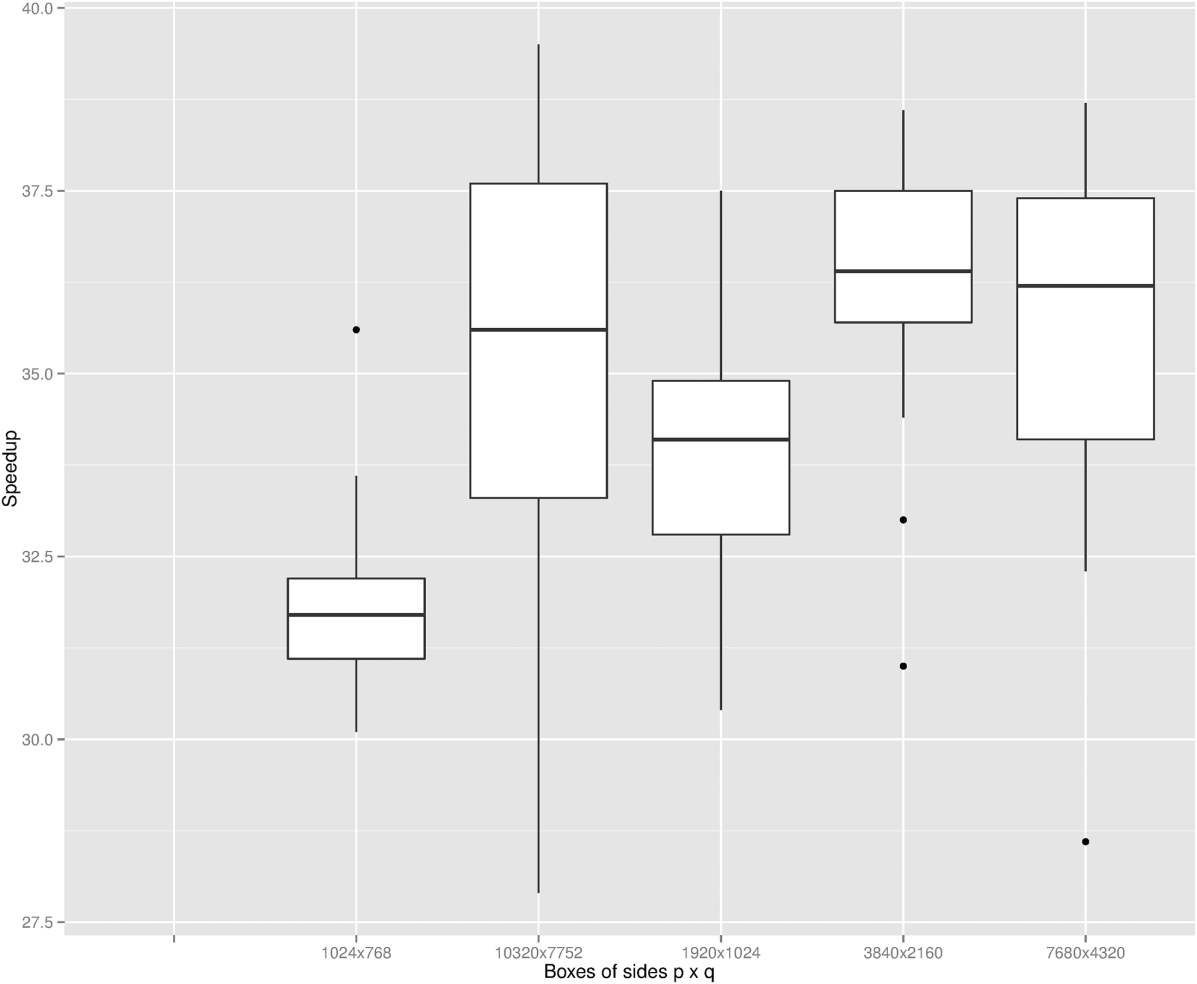
Speedup factor in OpenCV as a function of box size for a dense dataset. The points of each mammal in the datasete was first preconditioned with the method proposed here and then the convex hull was computed by OpenCV *convexHull* function.

For the case of *n* integer points inside a box of sides *p* × *q*, reducing the set to *s* points by the method proposed in this paper is empirically found to be better than reducing the points by the most common preconditioning method [8] under the condition *min*(*p*, *q*) ≤ *n*. This condition is typically satisfied in image datasets. In fact, all brain points of the typical image dataset used here are bounded in a box of size *p* × *q* = 50 × 256 pixels while *n* is roughly in the order of five thousand points. We used the *convexHull* function available within the open source computer vision library OpenCV to compute the hull for these images (from [11]). First, we applied the function to the original *n* points of an image and secondly to the remaining *s* points left by the preconditioning method to each image. We found an average speedup factor, due to the reduction in the number of points, of around a value of 1.9; the time it takes to apply the preconditioning was obviously taking into account. As we have used different box sizes to the dense dataset, we also quantified the speedup seen when using the *convexHull* OpenCV function on each mammals’ data. Speedup factors of over a value of 30 are clearly shown in Fig 7; they are rougly the same for each box size as the points in each box are very densely populated. For image datasets, we expect that checking the condition *min*(*p*, *q*) ≤ *n* is unnecessary and the precondition method can be directly applied to points (as points are pixels positions with integer values) for speeding up the convex hull computation.

## Conclusions

The reduced set of *s* points proposed here is recovered in such a way that a simple polygonal chain is formed from these *s* points. This allows the linear convex algorithm, known as Melkman, to be applied in a straightforward manner or any other known convex hull algorithm. When the condition *p* ≤ *n* holds for a dataset we have proved that the preconditioning method is of linear time *O*(*n*) and leaves *s* ≤ 2*p* points which results in a reduction of points by a factor of $1-\frac{2p}{n}$. The greater this reduction is, the greater the speedup factor observed for the computation of convex hulls in real datasets. In fact when the ratio *p*/*n* < 1/4 the reduction in the number of points is greater than half of the points; real datasets have *p*/*n* ratios much smaller and reductions of well over 95% were observed in the datsets used here. The preconditioning step in [8], proposed nearly four decades ago and still used and popular, typically reduces the number of points of datasets by around half the number of points; a much lower amount compared to the reduction achieved by the method proposed here. The proposed method differs from other methods in the sense that it can be thought of as a process of including points that may be in the final convex hull rather than a process of discarding points that cannot be part of the hull.

## Appendix

### Correctness of Algorithm 1

*Proof by induction* The claim is that the convex hull of the *n* original points contained in a box of size *p* × *q* points is the same as the convex hull obtained from the smaller set of *s* points contained in the output array *L* after the *n* points are processed by Algorithm 1. Asserting the claim proceeds by induction as follows.

The basis step is for *s* = 3 (after at least *n* = 3 points have been processed) since the smallest convex hull is a triangle. Three valid points are represented in array *L*. We distinguish, either case 1, three valid points found at positions *i* ≠ *k* ≠ *l*, or case 2, in which there are two valid points at position *i* and one valid point at position *k*, with *i* ≠ *k*, for any 1 ≤ *i*, *j*, *k* ≤ *p* for both cases. In case 1, clearly *n* = 3, so no reduction was achieved. In case 2, two valid points are found at (*i*, *u*) and (*i*, *v*) with *u* ≠ *v*; given that 1 ≤ *u*, *v* ≤ *q* implies that there could have been collinear points in between these two extreme valid points at position *i*. Thus, as the number of original points at position *i* was at most *q*, therefore *n* ≤ *q*+1, implying that Algorithm 1 reduces points (*s* < *n*) for any *q* > 2. By definition, any collinear point in between an edge of a convex hull cannot be part of the hull, so the claim holds. Assume, *L* contains *s* valid points, the claim holds in general if those *s* points capture the same convex hull of *n* ≥ *s* points. Given an array *L* with *s* valid points, consider adding an extra point within the limits of the bounding box. Three cases are now distinguished. Case 1, the new point is added at position *i* where no valid point previously existed. Case 2, the new point is added at position *i* where a single valid point previously existed. Case 3, the new point is added at position *i* where two valid points previously existed. In case 1, algorithm 1 does not make any assumption on removing it from the final convex hull and so a point (*x*, *y*) gets inserted into *L*[*x*] as (*y*, *y*); the claim holds. In case 2, *L*[*i*] had a valid point as (*u*, *u*) [or (*v*, *v*) since *u* = *v*]. The new point (*x*, *y*) gets inserted into *L*[*x*] either as (*u*, *y*) when *y* > *u*, or as (*y*, *u*) when *y* < *u* or remains as (*u*, *u*) when *y* = *u*; the claim holds in each instance. In case 3 either the new point (*x*, *y*) replaces an existing valid point in *L*[*x*] as minimum or maximum or it is discarded by being collinear to (*i*, *u*) and (*i*, *v*). In any instance either *n* = *s* or *n* > *s* (*n* = *s* + 1) and thus the claim holds.

## References

[1] Skiena S. The algorithm design manual (2nd ed.). Springer, London 2010.

[2] Furling JR, S.N. Majumdar SN, Comtet A. Convex hull of N planar brownian motions: exact results and applications to ecology. Phys. Rev. Lett. 2009;103(14):140602 10.1103/PhysRevLett.103.140602 19905556

[3] Dudok B, Barna L, Ledri M, Szabó SI, Szabadits E, Pintér B, et. al Cell-specific STORM super-resolution imaging reveals nanoscale organization of cannabinoid signaling. Nature Neuroscience. 2015;18:75–86. 10.1038/nn.3892 25485758 PMC4281300

[4] Klette G. Recursive computation of minimum-length polygons. Comp. Vision and Img. Understanding. 2013;117(4):386–392. 10.1016/j.cviu.2012.08.018

[5] Brassey CA, Sellers WI (2014). Scaling of Convex Hull Volume to Body Mass in Modern Primates, Non-Primate Mammals and Birds. PLoS ONE 9(3):e91691 10.1371/journal.pone.0091691 24618736 PMC3950251

[6] Preparata F, Shamos MI. Computational geometry: An introduction. Springer, London 1985.

[7] Melkman AA. On-line construction of the convex hull of a simple polygon. Info. Proc. Lett. 1987;25:11–12. 10.1016/0020-0190(87)90086-X

[8] Akl S, Toussaint G. A fast convex hull algorithm. Info. Proc. Lett. 1978;7:219–222. 10.1016/0020-0190(78)90003-0

[9] Cadenas J, Megson GM. Rapid preconditioning of data for accelerating convex hull computations. Elect. Lett. 2014;50(4):270–272. 10.1049/el.2013.3507

[10] CGAL. Available: http://www.cgal.org/.

[11] OpenCV. Available: http://www.opencv.org/.

[12] Chan TM. Optimal output-sensitive convex hull algorithms in two and three dimensions. Discrete and Computational Geometry. 1996;16:361–368. 10.1007/BF02712873

[13] Nordenskjöld R, Malmberg F, Larsson EM, Simmons A, Brooks SJ, Lind L, et. al Intracranial volume estimated with commonly used methods could introduce bias in studies including brain volume measurements. Neuroimage. 2013;83:355–360. 10.1016/j.neuroimage.2013.06.068 23827332

[14] Available: http://www.wri.org/resources/data-sets/kenya-gis-data/. Accessed 3 Sept 2015.

